# Perspectives on Anti-Black Racism and Mitigation Strategies Among Faculty Experts at Academic Medical Centers

**DOI:** 10.1001/jamanetworkopen.2022.8534

**Published:** 2022-04-22

**Authors:** Dedeepya Konuthula, Flor de Abril Cameron, Naudia Jonassaint, Eloho Ufomata, Orquidia Torres, Utibe R. Essien, Megan E. Hamm, Jessica Merlin, Maya I. Ragavan

**Affiliations:** 1 Internal Medicine–Pediatrics, University of Pittsburgh, Pittsburgh, Pennsylvania; 2 Division of General Internal Medicine, University of Pittsburgh, Pittsburgh, Pennsylvania; 3 Division of General Internal Medicine, University of Pittsburgh and University of Pittsburgh Medical Center, Pittsburgh, Pennsylvania; 4 Division of Adolescent and Young Adult Medicine, University of Pittsburgh and University of Pittsburgh Medical Center Children’s Hospital of Pittsburgh, Pittsburgh, Pennsylvania; 5 Division of General Academic Pediatrics, University of Pittsburgh and University of Pittsburgh Medical Center Children’s Hospital of Pittsburgh, Pittsburgh, Pennsylvania

## Abstract

**Importance:**

Black faculty and trainees remain underrepresented in academic medicine because of systemic racism. Years of diversity and inclusion efforts have not succeeded in eliminating the unique challenges faced by Black faculty in academic medicine.

**Objectives:**

To elicit expert faculty perspectives on anti-Black racism in academic medicine based on lived and/or professional experience and to solicit recommendations for an intervention for faculty to dismantle anti-Black racism within academic medical centers.

**Design, Setting, and Participants:**

This qualitative study included semistructured interviews with experts in understanding and dismantling anti-Black racism within academic medical centers. Participants had expertise in anti-Black racism through their lived experience as a Black faculty member and/or professional experience in diversity, equity, and inclusion efforts. Participants were recruited from academic medical centers from around the United States. Interviews were conducted through an online meeting platform, audio recorded, transcribed verbatim, and subsequently coded following an inductive qualitative description approach. Interviews were completed between October 2020 and March 2021.

**Main Outcomes and Measures:**

Outcomes include the experiences of Black faculty and trainees in academic medicine and recommendations for developing an intervention to dismantle anti-Black racism within academic medicine.

**Results:**

A total of 16 participants completed this study; most identified as Black or African American (9 [56%]) and female (10 [63%]). Results were sorted into 2 content domains, with several themes within those domains: (1) barriers faced by Black faculty and trainees and potential solutions and (2) recommendations for an intervention directed at faculty to dismantle anti-Black racism in academic medicine. Barriers faced by Black faculty and trainees included lack of representation; challenges with the recruitment, retention, and promotion of Black faculty; and experiences of microaggressions and overt racism. Participants suggested that an intervention should have a comprehensive learning objective; be mandatory for all faculty, with the exception of Black faculty; draw from outside expertise; and receive allocation of resources and funding equal to other important training modules.

**Conclusions and Relevance:**

The findings of this study affirm prior work about the unique challenges faced by Black faculty and trainees in academic medicine because of interpersonal and institutional racism and build on this prior work by soliciting recommendations to guide intervention development. An intervention to dismantle anti-Black racism within academic medicine is urgently needed and will require leadership buy-in and financial commitments from institutions for effective development and implementation.

## Key Points

**Question:**

What are the experiences of anti-Black racism among academic medical faculty, and what strategies are needed to address it?

**Findings:**

In this qualitative study including 16 experts in understanding and dismantling anti-Black racism, participants shared their perspectives on barriers and challenges faced by Black faculty and trainees as well as suggestions for a proposed comprehensive intervention for dismantling anti-Black racism in academic medicine.

**Meaning:**

These findings suggest that an intervention for anti-Black racism in academic medicine is needed urgently and will require leadership buy-in and financial commitments from institutions to be developed and implemented in an effective way.

## Teaser

This qualitative study presents expert faculty perspectives on anti-Black racism in academic medicine based on lived and/or professional experience and recommendations for an intervention for faculty to dismantle anti-Black racism within academic medical centers.

## Introduction

Black faculty and trainees remain severely underrepresented in academic medicine (AM) despite decades of diversity initiatives.^1,2,3^ Even when Black and other underrepresented in medicine (URM) students are admitted and matriculate to medical school, racial bias affects nearly every aspect of their assessment, with drastic consequences for their subsequent career opportunities, including their ability to be promoted, receive tenure, and be selected for leadership positions.^4,5,6,7,8,9,10,11,12,13,14,15,16,17,18,19^

To our knowledge, although a few older studies have examined the experiences of URM faculty, few have focused on anti-Black racism or reflect contemporary experiences of Black faculty.^20,21,22^ This gap in the literature is particularly important in light of increased institutional awareness of the need to support diversity, equity, and inclusion (DEI) efforts.^23,24,25,26,27,28,29^ Additionally, while studies of implicit and explicit bias training exist, we are not aware of any comprehensive interventions or curricula for faculty that target the many facets and effects of anti-Black racism in AM.^30,31,32,33^ Furthermore, little has been published on how leaders in the field of anti-Black racism envision an antiracist intervention for AM. Therefore, our objectives were to (1) elicit expert faculty perspectives on anti-Black racism in AM based on lived and/or professional experience and (2) identify preliminary targets for an intervention directed at faculty to dismantle anti-Black racism.

## Methods

We conducted semistructured interviews with experts in understanding and dismantling anti-Black racism in AM between October 2020 and March 2021. Data collection and analysis were supervised by a methodologist (M.E.H.) and conducted by members of a qualitative research group (Qualitative, Evaluation and Stakeholder Engagement research services [QualEASE]). The study team created the interview guide based on extant literature and the research team’s experiences. The guide comprised 2 sections, aligned with our objectives: (1) experience of Black faculty and trainees and (2) recommendations for developing an intervention directed at faculty to dismantle anti-Black racism within AM (eAppendix in the Supplement). The interview guide was pilot tested and revised by interviewing a member of our team (E.U.) who would have otherwise been eligible for inclusion. The University of Pittsburgh institutional review board deemed this study exempt from review, and all participants provided verbal informed consent. We did not follow the Consolidated Criteria for Reporting Qualitative Research (COREQ) reporting guideline prospectively but nevertheless included most of their recommendations.

### Reflexivity and Positionality of the Research Team

We carefully attended to reflexivity, ie, the impact of the research team on data collection, analysis, and interpretation.^34,35^ Three members of our team identify as Black (N.J., E.U., and U.R.E.), 1 as Black and Hispanic (O.T.), 3 as another minoritized racial and ethnic group (D.K., M.I.R., and F.d.A.C.), and 2 as White (J.M. and M.H.). Four (N.J., O.T., E.U., and U.R.E.) have expertise in anti-Black racism through both DEI leadership roles and lived experiences as Black faculty. Our team’s expertise also includes medical education leaders who have developed antiracism curricula (E.U., U.R.E., and O.T.) and researchers with expertise in health equity, community-based participatory research, and qualitative research (M.I.R., J.M., M.E.H., U.R.E., and F.d.A.C.). Our qualitative methods were designed to allow this diverse team to understand participants’ experiences and to ensure credibility of the analysis through the involvement of multiple coders, analysts, and team members in the analysis and subsequent discussion of the data.

### Participants and Recruitment

Eligibility criteria included self-identification as an expert in anti-Black racism within AM. We defined expertise as either professional (research or leadership), personal (lived experience as Black faculty in AM), or both. Informed by our collective experience, our team compiled a cross-disciplinary list of 31 eligible participants. Potential participants were contacted as many as 3 times by email. Twenty-four individuals responded; 16 agreed to participate, 1 declined to participate, and an additional 7 did not follow up.

### Data Collection

Given that the study occurred during the COVID-19 pandemic with interviewees across the United States, interviews were conducted and recorded remotely on an online meeting platform, with only the interviewer and interviewee present. The primary interviewer (F.d.A.C.) identifies as a cisgender woman and has been trained by the qualitative methodologist supervising the project (M.E.H.) with more than 10 years of interviewing experience working at QualEASE. F.d.A.C. knew 1 participant, who was interviewed by a different QualEASE interviewer. All interviews took approximately 60 minutes and were audio recorded and transcribed verbatim. Interviewees were also asked demographic questions including their title, self-identified race and ethnicity, self-identified gender, years working in AM, and discipline; participants could decline providing demographic information. Participants received a $100 gift card. We continued interviews until we reached thematic saturation, when no new codes or themes emerged during open coding.^35,36^

### Data Analysis

Analysis followed inductive qualitative description, a common approach in which analysts focus on describing the viewpoints of the interviewees as closely as possible without abstracting to the level of social theory.^37,38^ Two coders (F.d.A.C. and another trained QualEASE analyst) inductively created a codebook with a list of codes and their definitions. The final codebook consisted of 109 codes divided across several categories, including the experiences and manifestations of anti-Black racism, desired changes to address anti-Black racism, and categories related to the design and implementation of an anti-Black racism curriculum. They then coded 10 transcripts to demonstrate consistency. After adjudication, the primary coder (F.d.A.C.) coded remaining transcripts independently. Coding was completed with the assistance of Atlas.ti software. We conducted both conventional content and thematic analyses of the results, which were then shared with the broader study team for discussion and finalization of themes.^39,40,41^

## Results

Sixteen faculty participated, most of whom identified as Black or African American (9 [56%]) and female (10 [63%]) (Table). Participants represented 13 academic institutions and 11 states. Results were sorted into 2 content domains, with several themes within those domains. The Box includes more representative quotations.

**Table.** Demographic Characteristics of Participants

Faculty at academic medical centers	No. (%) (N = 16)^a^
Title	
Assistant professor	6 (38)
Associate professor	3 (19)
Director	2 (13)
Associate dean	1 (6)
Chair	1 (6)
Chief	1 (6)
Professor	1 (6)
Not reported	4 (25)
Race	
Black	
African American	9 (56)
Hispanic	1 (6)
Latinx	1 (6)
White or Asian Indian	1 (6)
Not reported	4 (25)
Gender	
Female	10 (63)
Male	1 (6)
Agender	1 (6)
Not reported	4 (25)
Academic medicine, mean (range), y	15.5 (4-41)
Disciplines	
Pediatrics	4 (25)
Internal medicine	3 (19)
Palliative care	3 (19)
Nephrology	1 (6)
Family medicine	1 (6)
Infectious disease	1 (6)
Emergency medicine	1 (6)
Hematology/oncology	1 (6)
Not reported	4 (25)

^a^ Percentages do not necessarily add up to 100% because of individuals with multiple titles or who work in multiple disciplines.

Box. **Domains, Themes, and Additional Representative Quotations**

**Domain 1: Barriers Faced by Black Faculty and Trainees and Potential Solutions**

**Theme 1: Lack of Representation**

“That then creates this whole cascade of, am I then representative of this stereotype … because there’s only one Black student versus if a White student was struggling.” (P 1)
“You’re going to be one of a small percentage of Black students in your class. So that initially just sets you up as the other, and often creates a target on the students to feel pressure to stand out, to be better, to be always on top of things, and to not have any mistakes or fail, for fear that they would be singled out even more than they already are. It’s just being the other.” (P 5)
“Black people see themselves and at the same time having to reconcile how others see Blacks. And so it’s this constant struggle of am I good enough? And … if I act this way … am I gonna make it bad for everyone behind me who’s Black?” (P 4)

**Theme 2: Recruitment, Retention, and Promotion of Black Faculty**

“Some of it ties I think specifically to the types of work that we often do that are not viewed as favorably or as highly among those who are doing the evaluations. So it’s kind of a tricky thing I’ll be honest because I think a lot of time Black faculty are evaluated differently not in a favorable way but then I think also there are some cases where Black faculty should be evaluated differently to create a more equitable process. That’s more so what I’m trying to get to. Recognizing that we tend to do different work that takes different amounts of time but maybe equally or more impactful you know how can we level the playing field so that it doesn’t adversely affect career trajectory.” (P 3)
“In the sense that if you did not represent your community you might have a better chance of being treated as a more worthy equitable faculty member but because you spoke up on behalf of that community you have sort of moved yourself out of the worthy zone into the less worthy zone because of what you represent—as opposed to saying well let’s talk about quality metrics.” (P 7)
“I do think that the work that’s done by Black faculty on diversity committees, on antiracist work, increasing diversity in learners should be something that counts towards promotion, something that money [and] protected time is put towards to encourage that as well.” (P 5)

**Theme 3: Microaggressions and Overt Racism**

“I think the experience is similar for faculty, but they have also learned how to guard against it. You know not all faculty in all medical centers wear a long white coat every day. But people who know that they’ve been mistaken for the cleaning staff or for the food delivery, they know to dress up the part: tie, white coat, you know not wearing scrubs more commonly.” (P 6)
“The smaller like microaggressions … that we see on a daily basis … those hit almost just as hard right because of the multiple daily insults. It was sad though, that we’ve come to a point where they get ignored on an interprofessional level because … it sometimes becomes a little bit exhausting to always be the one to call it out.” (P 8)

**Domain 2: Recommendations for an Intervention Directed at Faculty to Dismantle Anti-Black Racism in Academic Medicine**

**Theme 1: Learning Objectives of the Intervention Should Be Comprehensive**

“… [O]ne, having a better idea of what to say in the moment. ‘Cause I think in the moment if someone experiences some discrimination, I think everyone can be a little bit startled and can be confusing and uncertain how to respond in that situation. So, one, knowing how to respond in the situation. Two, knowing how to figure out what the person who experienced discrimination needs and how they’d benefit. And, three, not acting in a sense like a White savior, but as an advocate.” (P 12)
“People are going to need ongoing assessment and support to make sure that things are happening … talking about anti-Black racism, you can continue to … do anonymous surveys of your Black faculty and see are things getting better and if they’re not and what sort of things are continuing to happen, so that you can continue to focus your effort.” (P 14)

**Theme 2: Intervention Should Be Mandatory for All Faculty, With the Exception of Black Faculty**

“[The depression screen is] a new measure that we’re being measured on … some folks are like ‘Hey, I’m not a psychiatrist, I’m not necessarily focusing on that.’ … Our institution has decided that that’s an area that we will focus, right? And so we make sure that people get all of this training and then they have these measures and these goals and expectations. And I think that that’s how we should treat antiracism curriculum. That no, not everyone is going to be passionate or be interested in it to begin with, but if this is an important component of what makes us you know, truly, I don’t even want to say, excellent—I mean, just that makes us because I think it’s like a bare minimum.” (P 8)
“That maybe they don’t have to—well … I would say that maybe it has to be a choice, right? It doesn’t have to feel like it’s mandatory. And that’s another reason why I said, open it up to other groups. Including the other groups, then it doesn’t just put it on just the Black faculty members. Because you do bring up a good point. Like if someone comes to me and says, ‘Hey, [name of participant], I want you to get up in front of the department and talk about when you’ve been discriminated against,’ I’d be like, ‘I’d rather not.’” (P 10)

**Theme 3: Intervention Should Draw From Outside Expertise**

“It needs to be experiential, it needs to be more than an hour—I would suggest that it’d be more than one session. And that it be run by a diverse group two to three people at minimum, different sex, racial ethnic backgrounds, experiences, because that’s the kind of dynamic teaching that I think has the chance of the best effects.” (P 7)
“Another industry to look into is tech and business, and not to say that they’ve moved the needle that much further, but at least they have more of these training programs implemented at different companies.” (P 11)

**Theme 4: Structural Change Is Needed, Including Dismantling Power Inequities and Allocation of Resources and Funding**

“For the academicians that are there the Black ones, I would say promote them you know give them leadership titles, give them the training for them to be successful, give them the time to do their research, give them financial raises so they can support their families.” (P 4)
“I do think that the work that’s done by Black faculty on diversity committees, on antiracist work, increasing diversity in learners should be something that counts towards promotion, something that money [and] protected time is put towards to encourage that as well.” (P 5)
“They need bystander training or upstander training or intervention training so that when they see something or become aware of inequitable and abusive practices that disproportionately affect students of color that they know how to intervene and that they do. And then if they don’t, they need to be punished and I feel very strongly about this. … You have to say if you allow this to happen to one of your students you get pulled out of that environment and you have negative consequences because you had a power position and you did nothing to protect them.” (P 7)

Abbreviation: P, participant.

### Domain 1: Barriers Faced by Black Faculty and Trainees and Potential Solutions

#### Theme 1: Lack of Representation

Black-identifying participants described struggling with visibility: “to be Black or a person of color is to be both … hyper-visible and also invisible at the same time … because numbers are so small” (Participant [P] 1). Several participants described how limited representation causes Black students, trainees, and faculty to feel that they are not allowed to struggle because of stereotype threat and the way that weakness is attributed to inferiority based on race: “Whereas this African-American trainee is second guessing themselves … is that part of stereotype threat and feeling that perhaps I’m not as good as I think I am or just feeling like they have to hold the weight of the entire race” (P 8).

#### Theme 2: Recruitment, Retention, and Promotion of Black Faculty

Participants argued that Black faculty recruitment, retention, and promotion challenges contribute significantly to lack of representation. One participant described how Black students are impacted by medical school acceptance policies: “we know that structural racism impacts Black people from infancy, and so there are so many hurdles and challenges. … It is not as likely that they all end up at the Harvards and the Ivys of the world and so that makes it more challenging for them to get into medical school” (P 16). Another participant described how long-term retention may be a challenge even when recruitment is successful: “they get these big classes of residents that have a higher number of Black residents or Black trainees or early career Black faculty and then they don’t really do anything with them after that. They don’t really treat them in the same way—the way they did to recruit them” (P 2).

Many participants also described the minority tax, which involves “saddling the faculty member to do all these things [related to DEI]. And then you know down the line they are asking where are your papers, where are your grants, and if you don’t have those things, then you’re going to have to do more clinical time to make up your salary. And not acknowledging that they’ve been given all these other tasks that are not recognized” (P 4).

However, many participants felt that DEI work is an important part of the mission of AM and discussed ways that amplifying the value of DEI work within academia is critical. One participant shared they felt the push behind DEI work “really has to start from the top of the organization in terms of having invested leaders thinking about this, actively seeking out to create this environment, giving the resources necessary to make it happen [instead of making it] the responsibility of a few underrepresented in medicine faculty, fellows, trainees to really do all this work that’s not compensated, it’s not rewarded, not recognized” (P 2).

#### Theme 3: Microaggressions and Overt Racism

Participants described how Black students, trainees, and faculty are expected to function at a high level in an environment where they are forced to contend with both overt racism and microaggressions. One participant explained that “there are certain instances where I think that’s a blatantly racist statement where unfortunately no one really felt empowered to stand up and clarify ‘what exactly does that mean?’” (P 8).

The importance of accountability and creating mechanisms for reporting racist offenses with transparency regarding the consequences was also stressed. “I think when you put your money where your mouth is … people tend to straighten up you know—when you have financial disincentives or career disincentives then people will start to make a change. Even if they don’t want to” (P 4).

### Domain 2: Recommendations for an Intervention Directed at Faculty to Dismantle Anti-Black Racism in Academic Medicine

#### Theme 1: Learning Objectives of the Intervention Should Be Comprehensive

Participants underscored the need to ensure faculty have a solid foundation on the historical underpinnings of anti-Black racism and how policies within their institution lead to disparities. One participant stated: “they would understand the terminology around racism, the definitions and then how that gets applied to the world and academic medicine. I’m thinking implicit bias, structural racism, equity—those sorts of things, and I would also think an objective would be … to gain a better understanding of how racism impacts Black faculty specifically in academic medicine and to walk away with ways to implement and advocate for change” (P 2).

Interviewees emphasized the importance of practicing antiracism skills similarly to learning other parts of clinical medicine: “we need to practice with cases and with direct observation— watching people do it the right way and giving them feedback about how it went” (P 6). Another participant highlighted the importance of self-reflection: “There has to be a lot of self-inner work that I don’t think people in medicine do very well. … A lot of time for self-reflection and understanding why these behaviors exist, why you may demonstrate these behaviors yourself and you don’t really want to come to terms with it … more mindfulness practice, more restorative justice” (P 11). Participants noted how the intervention should be comprehensive and go beyond implicit bias training: “Not like a two-hour unconscious bias training, like a really deep dive into trends, into experiences, real experiences of bias, into role-playing if that’s needed” (P 4).

#### Theme 2: Intervention Should Be Mandatory for All Faculty, With the Exception of Black Faculty

Participants strongly felt that training should be mandatory: “…why is sexual harassment training mandatory? Why is CITI [Collaborative Institutional Training Institute] training for IRB [institutional review board] research? It’s because it’s important. Mandatory to a certain extent says that this is valued or this is important for some reason” (P 1). Some participants highlighted the importance of training leaders because “if you’re in the leadership position, like if you’re hiring people or you’re a chair of a department, I think there needs to be specific education for those people in terms of how to reduce bias in the hiring process, or how to defuse situations, or how to address a staff member or a faculty member who is being racist or who has been brought to attention because of concerns about their own racism towards their colleagues or towards patients” (P 5). Participants also highlighted that leadership must be invested and make it known that participation is expected: “It is really hard to get faculty members to commit, or learners or anyone to commit when the leaders themselves haven’t done the work” (P 8).

Participants held different opinions about whether Black students and faculty should participate. One interviewee suggested that required training might be harmful and retraumatizing if not done thoughtfully: “Black friends and colleagues and others have been part of different trainings or experiences and it’s been very frustrating and hurtful for them. … It ends up being more emotionally traumatizing for them than helpful” (P 1). Other participants thought that Black faculty could benefit since “you can’t take for granted that just because you’re [a] Black faculty member that you know everything there is to know about racism and how to tackle it” (P 8). Some participants suggested that Black faculty could opt out of certain aspects of training, such as sessions that delve into the basics of anti-Black racism or decline to offer thoughts around a particular topic: “I kind of can’t emphasize enough that I’ve heard from lots of folks particularly this year that they’re just exhausted and they’re sort of tired of talking about it” (P 3).

#### Theme 3: Intervention Should Draw From Outside Expertise

Some participants suggested leveraging the expertise of disciplines both inside and outside of academia: “I think you might have to go to sociology and anthropology, social work. … There are people who are experts in critical race theory. If you’re wanting the educators to be healthcare clinicians, you may have to start first with getting them educated, right? And then you probably need to talk to people in education” (P 13). Another participant noted: “there [are] real companies that work and consult and go into Fortune 500 companies and tell them what they need to do to be more inclusive and to not be racist. I think academia needs to do the same” (P 4).

#### Theme 4: Structural Change Is Needed, Including Dismantling Power Inequities and Allocation of Resources and Funding

Proposed structural changes included promotion of Black faculty to leadership positions, comprehensive review of institutional policies, and accountability for individuals who perpetuate anti-Black racism. As one participant noted, “it also would look like White people and non-Black other minorities stepping back from prominent spaces to make space for Black academic leaders” (P 6). Another participant shared: “We need to get rid of the people who are perpetuating anti-Black racism. … It needs to be an external group that investigates incidences of discrimination. … If there are proven incidences of discrimination, those people need to lose their jobs. This needs to be no longer a slap on the wrist, a university wide email saying we shouldn’t do this anymore and unconscious bias training, we’re beyond that” (P 4).

Participants also emphasized that the work of developing, implementing, and facilitating an intervention requires monetary and time compensation: “What you value is what you put your money behind. … Any large change requires a capital investment, an investment in people. … To ask people to do this without that or to ask them to do it just as an aside, then what you will get is a nice program that you will publish that nobody else implements, and nobody’s life is any better” (P 13).

## Discussion

Our study describes multiple challenges faced by Black faculty and trainees consistent with research from a decade ago, including lack of representation, hypervisibility and invisibility, limited opportunities for recruitment and promotion, experiences of racism and discrimination, and a lack of institutional support.^19,20,21,22,24,42,43,44,45,46,47^ Our reaffirmation of this prior work demonstrates how, disappointingly, little progress has been made and highlights the critical need for anti-Black racism interventions in AM. We take an important next step by offering sustainable solutions and identifying potential intervention targets.

The minority tax was amplified more in this study than in prior work.^20,21,22^ This may be because institutions have recently approached DEI work with greater urgency, which can burden Black trainees and faculty who are often not properly compensated or promoted.^24,48,49^ Effective interventions will require investment. Leadership—the majority of whom are not Black—must have an enriched understanding of the racist barriers to advancement in AM and work with intentionality to promote students, trainees, and faculty belonging to minoritized racial and ethnic groups.^17,50,51^

Despite deeply rooted anti-Black racism permeating AM, participants were not aware of comprehensive interventions for faculty. Many AM centers offer implicit bias training; however, our study underscores that an effective antiracist intervention must be more comprehensive. There are some existing interventions for clinicians as well as the Academics for Black Survival and Wellness Anti-Racism Training, which is broadly applicable.^52,53,54^ However, AM is a unique space that exists at the intersection of clinical care, education, and research and as such requires a targeted intervention that addresses how anti-Black racism permeates each of these components. Our study also underscores the complexity inherent in mandating antiracism training for Black faculty and highlights that including Black faculty in an interactive educational intervention could burden, retraumatize, and create pressure to speak on behalf of other Black colleagues. When designing this intervention, it will be crucial to be mindful of which components may be helpful for Black faculty as well as those that may be detrimental.

### Implications

This work sets a foundation for developing an intervention for anti-Black racism in AM. Next steps for research may include gathering recommendations from experts in other fields, including education and sociology, as well as from trainees, patients, staff, and community organizations. Surveying the perspectives of leaders in AM, particularly given that our study focused on assistant and associate professors, would also be useful. We will also need to cocreate the intervention with important partners and secure institutional commitments of funding, resources, and support toward the development and implementation of a comprehensive intervention.

### Limitations

We recognize several limitations to this study. We drew participants from our networks, which may risk missing other varied perspectives, eg, a broad range of clinical and surgical subspecialties. We also limited our study to faculty; future research should amplify the voices of other critical individuals in AM such as trainees, patients, clinical staff, and research staff. Our overall number of participants was small; however, 16 participants is an adequate sample to reach 90% thematic saturation and allowed our team to reach saturation.^36^ Expertise was self-identified; however, our initial list of participants was developed through the joint networks of our research team, many of whom are themselves experts in anti-Black racism and health equity. Furthermore, we focused our study on developing an intervention at the faculty level, as faculty play multiple roles in AM, have a sustained presence in AM, and have the responsibility of creating and safe and supportive learning environment. However, we recognize that anti-Black racism interventions should also be developed for and by learners (medical students, residents), staff, leadership, and other key groups in AM.

## Conclusions

The deleterious effects of anti-Black racism on trainees and faculty in AM have been well-documented for over a decade. AM has a moral imperative to develop comprehensive interventions that dismantle anti-Black racism and promote the thriving of Black-identifying members of our communities.

## Notes

Supplement. **eAppendix.** Interview Guide
