# Error in Author Affiliations

**DOI:** 10.1001/jamanetworkopen.2022.18888

**Published:** 2022-06-01

In the Original Investigation titled “Perspectives on Anti-Black Racism and Mitigation Strategies Among Faculty Experts at Academic Medical Centers,”^1^ published April 22, 2022, there was an error in the author affiliations. Dr Merlin’s affiliation should have been Division of General Internal Medicine, University of Pittsburgh and University of Pittsburgh Medical Center, Pittsburgh, Pennsylvania. This article has been corrected.^1^
