# Supplementary material for: Perspectives on Anti-Black Racism and Mitigation Strategies Among Faculty Experts at Academic Medical Centers
Source: JAMA Netw Open. 2022 Apr 22;5(4):e228534. doi: 10.1001/jamanetworkopen.2022.8534 (PMC9034397; doi:10.1001/jamanetworkopen.2022.8534)
Supplement: Supplement. — eAppendix. Interview Guide [file jamanetwopen-e228534-s001.pdf]

Supplemental Online Content

Konuthula D, de Abril Cameron F, Jonassaint N, et al. Perspectives on anti-Black racism and mitigation strategies among faculty experts at academic medical centers. *JAMA Netw Open.* 2022;5(4):e228534. doi:10.1001/jamanetworkopen.2022.8534

eAppendix. Interview Guide

This supplemental material has been provided by the authors to give readers additional information about their work.

© 2022 Konuthula D et al. *JAMA Network Open.*

eAppendix. Interview Guide

Thank you for taking the time to talk with us today. In this study, we will ask you questions about racism in academic medicine experienced by Black faculty, students, and staff. Our specific goal is to develop a curriculum for faculty in academic medical centers focused on rejecting anti-Black racism and promoting respect and understanding of the Black community.

Introductory question: Please describe your role in academic medicine

When you hear the phrase "anti-Black racism in academic medicine," what comes to mind?

> Prompt if needed: What do you think anti-Black racism in academic medicine looks like?

How can we restructure academic medicine so that it eliminates anti-Black racism for faculty at academic medical centers? Please describe what you would do.

> Please describe policy changes needed to reject anti-Black racism in academic medicine

> Please describe educational changes needed to reject anti-Black racism in academic medicine

<u>Specific intervention questions</u>

We are interested in learning about interventions for non-Black faculty to enable them to be anti-racist advocates. Tell me about this idea.

In the next few questions, I will be asking for your opinion about how to develop and implement such a program.

Note to the interviewer: academic medicine is broad and can include patient care, education, research, administrative, and other. Depending on the role of the participant, may need to probe/adapt as need.

Please describe the key learning points and skills that you would hope faculty would gain after participating in such a program.

Please described the topics that should be included in such a program? What do you think is most important to teach?

Please describe who should participate in this intervention?

> Probe: Some interviewees thought Black faculty should not be part of this intervention, others thought they should. What are your thoughts?

Who should facilitate this program?

© 2022 Konuthula D et al. *JAMA Network Open.*

Probe: Black faculty, White faculty, leadership, someone else?

Who should train the facilitators?

How can institutions assign value to the work of facilitating this intervention?

How do we reach individuals who may not otherwise participate in the intervention?

What do you think might generate buy-in for non-Black faculty?

What do you think will help non-Black faculty feel confident in their anti-racism skills?

How can faculty practice and get feedback on the skills they learn?

Please describe how non-Black faculty should be involved in diversity and inclusion efforts?

In some of the interviews we have done so far, we have heard that a possible intervention could look like a series of workshop, for all faculty, center on understanding histories of racism and restructuring medicine, and focus on incorporating tangible skills. We would love if you could reflect on this feedback.

Many of the interviews have focused on interventions aimed for clinician educators. Are there different topics that clinician investigators or clinician leaders should learn?

Conclusion questions

Before we end, is there anything else I didn't ask you which you think is important and related to this topic?

© 2022 Konuthula D et al. *JAMA Network Open.*
